# Sodium-Glucose Cotransporter-2 (SGLT2) Inhibitors as Therapy for Hidradenitis Suppurativa and Other Inflammatory Skin Diseases: A Narrative Review

**DOI:** 10.7759/cureus.110202

**Published:** 2026-06-03

**Authors:** Declan C McCann, Ben Martinez, Nathalia G O’Brien, Harvey N Mayrovitz

**Affiliations:** 1 Medicine, Nova Southeastern University Dr. Kiran C. Patel College of Osteopathic Medicine, Davie, USA; 2 Medical Education, Nova Southeastern University Dr. Kiran C. Patel College of Allopathic Medicine, Davie, USA

**Keywords:** cytokines, hidradenitis suppurativa, immunometabolism, inflammasome, inflammation, insulin resistance, metabolic syndrome, nlrp3, sglt2i, sodium-glucose cotransporter-2 inhibitor

## Abstract

Hidradenitis suppurative (HS) is a chronic inflammatory skin disorder characterized by deep-seated nodules, abscesses, sinus tract formation, and scarring. Growing evidence has begun to support the notion that HS not only is a local follicular disorder but also has strong associations with metabolic dysfunction. The metabolic abnormalities seen in patients with HS include obesity, insulin resistance, metabolic syndrome, adipose-mediated inflammation, and altered cytokine signaling. Sodium-glucose cotransporter-2 inhibitors (SGLT2is) are a class of drugs currently used to lower blood glucose in patients with type 2 diabetes mellitus (T2DM). However, recent studies have provided evidence that SGLT2i can alter multiple immunometabolic mediators, including reducing nucleotide-binding oligomerization domain (NOD)-like receptor protein 3 (NLRP3) inflammasome activity, lowering pro-inflammatory cytokine levels, and improving insulin resistance. The purpose of this review was to determine whether SGLT2i can alter mechanisms involved in the development of HS through their anti-inflammatory and metabolic effects. Using Web of Science and PubMed, a literature review was conducted to compile studies on HS, metabolic dysfunction, and the anti-inflammatory effects of SGLT2is. The current evidence indicates that the metabolic and inflammatory pathways targeted by SGLT2is contribute to the development of HS, including the NLRP3 inflammasome, cytokine production, and adipose-related inflammation. The evidence supporting this is largely mechanistic; therefore, future clinical studies are needed to determine whether SGLT2i use in HS can provide significant improvement. However, with current therapies targeting downstream inflammatory mediators, SGLT2i and potentially other metabolic therapies may be promising adjunctive therapies by targeting upstream metabolic drivers that amplify these mediators.

## Introduction and background

Hidradenitis suppurativa (HS) is a chronic inflammatory skin disorder with a prevalence of 0.1%-1% that is characterized by several clinical manifestations, including deep-seated nodules, abscesses, sinus tract formation in intertriginous areas, and scarring [1,2]. It is associated with significant physical and psychosocial morbidities and remains challenging to manage with current available therapies, often requiring multiple modalities [2-4]. Current therapies range from antibiotic regimens to biologic therapies, such as interleukin 17 (IL-17) (secukinumab and bimekizumab) and tumor necrosis factor-alpha (TNF-α) (adalimumab) inhibitors, to surgical intervention depending on the severity of the disease [5]. HS has historically been viewed as a localized follicular disorder; however, recent studies have provided evidence that suggests the condition represents a systemic inflammatory disorder with strong metabolic associations [6,7]. ​Epidemiologic studies have consistently shown that patients with HS exhibit worse metabolic profiles than the general population, including higher rates of obesity, insulin resistance, type 2 diabetes mellitus (T2DM), and metabolic syndrome [6]. These findings suggest shared pathophysiological drivers among metabolic dysfunction, immune dysregulation, and HS, rather than just HS itself. It is suggested that systemic, low-grade chronic inflammation and dysregulated adipokine profiles link metabolic dysfunction and immune activation in HS [6-8].

​At the molecular level, HS lesions have been found to have higher levels of the nucleotide-binding oligomerization domain (NOD)-like receptor protein 3 (NLRP3) inflammasome, IL-1β, IL-6, IL-17, and TNF-α, implicating their involvement in the development of the disease, suggesting that inflammasome activation and cytokine signaling play important roles in driving HS inflammation [7,8]. Insulin resistance and hyperinsulinemia are known to activate many of these cytokine pathways through effects on immune cells, cytokine production, and keratinocyte proliferation [9-11]. These overlapping mechanisms suggest that therapies targeting metabolic pathways may benefit HS. Sodium-glucose cotransporter-2 inhibitors (SGLT2is) are an established treatment for T2DM that work by inhibiting sodium-glucose cotransporters in the proximal tubules, leading to glucosuria [12]. However, other than glycemic control, SGLT2is have been found to have metabolic and anti-inflammatory benefits, including lowering insulin resistance, NLRP3 inflammasome activity, and adipose-mediated inflammation and cytokine production [13-15].

Despite the growing evidence of HS being a systemic inflammatory disorder with strong metabolic links, current available therapies are targeted at the downstream inflammatory mediators, leaving the possibilities for therapies aimed at the metabolic relationship relatively understudied. Only one study has investigated the direct effects of SGLT2i use in HS; however, this study focused on SGLT2i’s ability to reduce vascular risk and mortality in patients with HS and not on modifying the disease itself [16]. Given the overlap among HS, metabolic dysregulation, inflammasome activation, and cytokine signaling, it is important to investigate whether therapies that improve metabolic profiles can also have a beneficial impact on disease pathophysiology. Therefore, the purpose of this narrative review was to synthesize evidence to address the following research question: do SGLT2i, through their metabolic and anti-inflammatory effects, have the potential to alter key mechanisms in the pathogenesis of HS and serve as a possible therapeutic approach?

## Review

Methods

Search Methodology

PubMed and Web of Science electronic databases were comprehensively searched for relevant articles bearing on the pathophysiology of HS and the overall systemic effects of SGLT2i. Papers published from January 2010 to February 2026 were considered in the search.

Search Strategy

The search was conducted using the following combination of terms: (“HS” OR “hidradenitis suppurativa”) AND (“SGLT2 inhibitors” OR “sodium-glucose cotransporter-2” OR “biologics”) AND (“metabolic syndrome” OR “obesity” OR “immunometabolism” OR “NLRP3 inflammasome” OR “cytokines” OR “IL-1B” OR “IL-6” OR “IL-17” OR “TNF-a” OR “inflammation” OR “type 2 diabetes mellitus” OR “T2DM” OR “mechanism”). Additionally, these combinations were supplemented with pharmacologic and mechanistic terms including “dapagliflozin,” “empagliflozin,” “canagliflozin,” “ketone bodies,” “B-hydroxybutyrate,” “macrophage polarization,” and “adipokines.” Also included were search terms for inflammatory skin diseases with inflammatory markers similar to those of HS, including “psoriasis” and “alopecia areata.”

Inclusion Criteria

Inclusion criteria covered studies that were peer-reviewed and published in English and reported on the following: the pathophysiology of HS, metabolic or inflammatory mechanisms associated with HS, psoriasis, alopecia areata, pharmacologic effects of SGLT2i on metabolic or immune pathways, and studies that evaluated SGLT2i in inflammatory or metabolic diseases relevant to HS. Studies that dealt with these issues in humans, animals, or in vitro were considered.

Exclusion Criteria

Excluded from consideration were abstracts, case reports, small case series with fewer than 10 subjects, and articles published prior to 2010.

Data Extraction and Presentation

Because the review is a focused narrative design, no risk-of-bias assessment or quantitative synthesis was conducted, nor was any statistical synthesis or meta-analysis performed. The data were extracted qualitatively and organized into thematic sections, including pharmacology and systemic effects of SGLT2i; pathophysiology and metabolic links of hidradenitis suppurativa; evidence supporting SGLT2i use in hidradenitis suppurativa; evidence supporting SGLT2i use in other inflammatory skin diseases, including psoriasis and alopecia areata; clinical implications and translational potential; and safety considerations relevant to dermatology. As a result, the heterogeneity of the included studies may limit the ability to draw definitive conclusions. Additionally, this review was not conducted using a predefined protocol or registration, which may limit full methodological reproducibility.

Results

**Figure 1:**
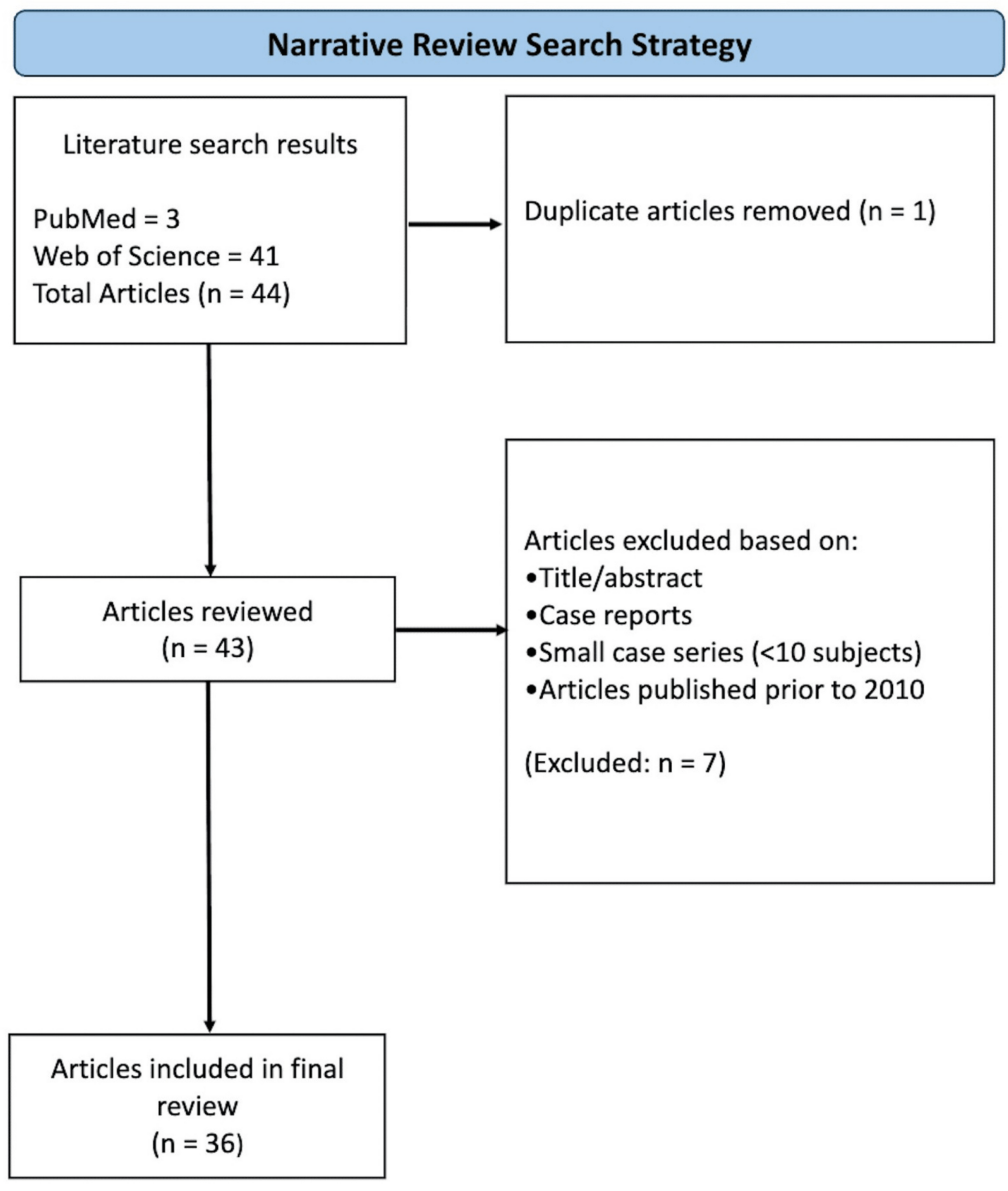
Article selection process for the narrative review. Flowchart illustrating the search results, review, and inclusion of studies from PubMed and Web of Science. A total of 44 articles were identified, one duplicate was removed, 43 records were reviewed, and 36 studies were included based on relevance to HS, immunometabolic mechanisms, and effects of SGLT2i. Image credits: Nathalia G. O’Brien. Created using Microsoft PowerPoint (Microsoft Corp., Redmond, WA). No AI-assisted tools were used. HS, hidradenitis suppurative; SGLT2i, sodium-glucose cotransporter-2 inhibitor

A total of 44 articles were identified during the search. After the removal of duplicates (n=1), 43 titles were reviewed, and 36 titles were included to address the mechanistic pathways, pharmacologic effects, and clinical associations relevant to HS and SGLT2 inhibition. The study selection process is summarized in Figure 1.

Pharmacology and Systemic Effects of SGLT2 Inhibitors

SGLT2is act within the renal proximal tubule by inhibiting sodium-glucose cotransport, causing glucosuria and lower blood glucose, independent of insulin [12]. By doing so, SGLT2is prevent hyperglycemia and lower both fasting serum insulin and homeostatic model assessment for insulin resistance (HOMA-IR) [15,17]. Additionally, treatment with SGLT2i has been found to be associated with a shift in metabolic pathways toward lipid oxidation, reflected by increased circulating β-hydroxybutyrate (BHB), and other ketone bodies, free fatty acids, and reduced fasting serum insulin [17]. Collectively, these findings indicate a metabolic reprogramming rather than just glucose lowering. This is important because these metabolic changes can reduce upstream promoters of inflammation, linking glucose and lipid metabolism to immune pathway modulation in HS.

​Importantly, SGLT2is have systemic anti-inflammatory effects relevant to chronic inflammatory diseases. Clinical studies performed on patients with type 2 diabetes mellitus (T2DM) found that SGLT2is reduced circulating levels of the interleukins IL-1β and IL-6 and TNF-α and increased the level of anti-inflammatory cytokine IL-10 [18]. At the cellular level, SGLT2 inhibition suppresses NLRP3 inflammasome activity and, therefore, reduces IL-1β expression [18]. Experimentally, in vivo studies found a shift of macrophage polarization from M1 to M2. This shift is significant, as it represents a transition from a pro-inflammatory (M1) phenotype to an anti-inflammatory (M2) phenotype. There is also a decrease in nuclear factor kappa-light-chain-enhancer of activated B cells (NF-κB) activation and toll-like receptor 4 (TLR4) expression [18]. These changes are significant because both NF-κB and TLR4 play key roles in activating the innate immune system, and by suppressing them, SGLT2i can decrease the transcription of pro-inflammatory cytokines. Taken together, these findings support SGLT2i as an immunometabolic modulator with systemic effects other than glycemic control. This is important because it shows that SGLT2i can modulate metabolic and immune pathways by targeting upstream mechanisms that drive chronic inflammation in HS.

Together, these findings show that SGLT2is affect both metabolic and inflammatory pathways that play major roles in the pathogenesis of HS. SGLT2i can inhibit upstream drivers of chronic inflammation by improving insulin resistance, shifting metabolism toward lipid oxidation, and suppressing inflammatory mediators, including the NLRP3 inflammasome and proinflammatory cytokines. This broad effect on inflammatory pathways demonstrates the potential for SGLT2i to be used in the treatment of HS.

Pathophysiology and Metabolic Links of Hidradenitis Suppurativa

HS is a chronic inflammatory disorder of the folliculopilosebaceous unit that is characterized by nodules, abscesses, sinus tracts, and scarring in intertriginous skin [1]. It is thought to begin with the hyperkeratinization of the follicular lining, which leads to follicular occlusion with keratin fibers, hair fragments, bacteria, and pathogen-associated molecular patterns/damage-associated molecular patterns (PAMPs/DAMPs) [1,19]. Eventually, this causes the follicle to rupture and release its contents, activating the innate immune system, including toll-like receptors (TLRs) and the NLRP3 inflammasome [19]. The activation of NLRP3 induces caspase-1 to process pro-IL-1β and pro-IL-18, triggering a cascade of inflammatory signaling [19]. IL-1β plays a major role in HS through the recruitment of neutrophils, the promotion of matrix metalloproteinase (MMP) production, and the triggering of T helper 17 cell (Th17) differentiation, resulting in elevated levels of IL-17, TNF-α, and IL-6 [19,20]. These cytokines maintain chronic inflammation and contribute to the formation of sinus tracts and persistent lesions. This highlights that HS is driven by abnormal innate immune activation and the amplification of inflammatory cytokines following the rupture of a plugged follicle-lipid-sebaceous unit, leading to persistent chronic inflammation and tissue destruction.

​Growing evidence has also linked HS to systemic metabolic dysfunction. Both obesity and metabolic syndrome have been found to be highly prevalent in HS and share common inflammatory mechanisms, including chronic low-grade inflammation and dysregulated adipokine signaling [6,8,21]. Adipose tissue functions as an endocrine organ, releasing cytokines and adipokines that can amplify the inflammatory pathways that are involved in HS, particularly TNF-α and IL-6 [8]. Additionally, patients with HS have been found to have higher rates of insulin resistance and metabolic abnormalities compared to controls [22]. Inflammatory cytokines, such as TNF-α and IL-6, are capable of impairing insulin signaling, contributing to systemic metabolic dysfunction, further reinforcing the inflammatory pathways involved in HS [22]. This demonstrates that metabolic dysfunction directly contributes to the pathogenesis of HS by sustaining inflammatory signaling and maintaining a chronic inflammatory state.

Together, the above findings are consistent with HS being caused by follicular structural abnormalities, along with innate immune activation and systemic metabolic dysfunction. This would then link HS pathogenesis to pathways targeted by metabolic therapies.

Evidence Supporting SGLT2i Use in HS

Building on the pharmacologic mechanisms described above, SGLT2i may have the ability to influence several pathways involved in the pathogenesis of HS. There are currently limited clinical trials that have investigated the use of SGLT2i in HS; however, there is a great deal of mechanistic evidence that this drug class can target many key pathways implicated in HS pathogenesis. One such pathway is the NLRP3 inflammasome pathway, which SGLT2is have been shown to attenuate. The activation of the NLRP3 inflammasome plays a key role in HS by promoting downstream inflammatory signaling and Th17-mediated immune response [7]. Other key pro-inflammatory cytokines involved in the pathogenesis of HS, such as IL-1β, IL-6, IL-17, and tumor necrosis factor-alpha (TNF-α), have been shown to decrease with the use of SGLT2is [7,8]. Strategies that aim to reduce the NLRP3/IL-1β signaling may have potential therapeutic effects in treating HS [7,8].

Both clinical and experimental studies have shown that SGLT2is exert inhibitory effects on NLRP3 inflammasome activity. Human models have shown that this therapy reduces IL-1β production, suggesting that this agent may directly act on key pathways relevant to HS pathogenesis [17,18]. As described previously, the shift toward lipid oxidation and ketone body production induced by SGLT2i may contribute to suppressing NLRP3 inflammasome activity in HS [16]. This mechanism provides a potential link between SGLT2i metabolic changes that cause downstream anti-inflammatory effects [17].

In addition to their metabolic effects, SGLT2i use has been associated with reductions in pro-inflammatory cytokines and an increase in anti-inflammatory signaling pathways [18]. It is likely that the use of SGLT2i shifts the body toward an anti-inflammatory state. Because these same inflammatory markers play critical roles in the pathogenesis of HS lesion inflammation, their suppression gives reason for further investigation in using SGLT2i as therapeutic agents in HS [7,8].

SGLT2i may influence immune function by modulating macrophage polarization. The use of SGLT2i in experimental models has shown a shift in macrophage polarization from the M1 phenotype to the anti-inflammatory M2 phenotype, further supporting the role that SGLT2is play in promoting an anti-inflammatory state [18]. Alongside this shift in polarity, there is a reduction in NF-κB signaling [18]. The modulation of macrophage signaling is another potential mechanism by which SGLT2i may reduce inflammation and HS pathogenesis.

There are also metabolic associations of patients with HS, frequently exhibiting insulin resistance, obesity, and metabolic syndrome [6]. SGLT2is have direct consequences on all these metabolic factors. HOMA-IR is a measure of insulin sensitivity and is improved in patients on SGLT2i [15]. SGLT2i increases fat oxidation and ketone body levels, which confer metabolic anti-inflammatory effects. Low-grade chronic inflammation, adipose dysfunction, and insulin resistance may all be improved with these therapeutic agents.

**Figure 2:**
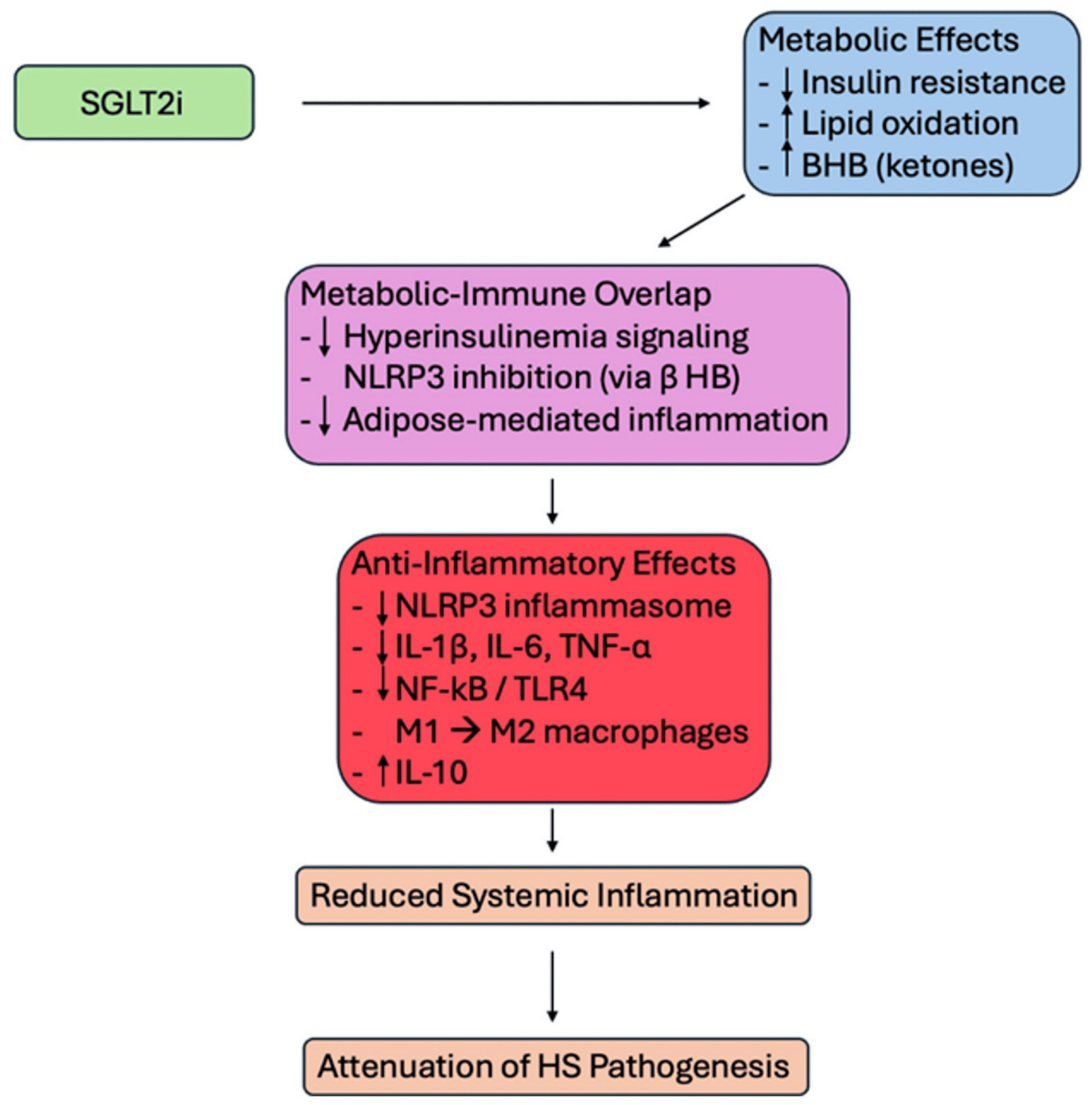
Proposed immunometabolic mechanisms by which SGLT2is attenuate inflammation in hidradenitis suppurativa (HS). Sodium-glucose cotransporter-2 inhibitors (SGLT2is) induce systemic metabolic changes, including reduced insulin resistance, increased lipid oxidation, and elevated ketone body (β-hydroxybutyrate, BHB) levels. These metabolic effects intersect with immune signaling pathways, leading to decreased hyperinsulinemia-driven signaling, the inhibition of the NLRP3 inflammasome (partially mediated by BHB), and reduced adipose tissue-associated inflammation. Collectively, these changes exert anti-inflammatory effects, including the suppression of NLRP3 inflammasome activity, the decreased production of pro-inflammatory cytokines (IL-1β, IL-6, and TNF-α), and the attenuation of NF-κB and TLR4 signaling pathways. Additionally, SGLT2 inhibition promotes macrophage polarization from a pro-inflammatory M1 phenotype toward an anti-inflammatory M2 phenotype and increases IL-10 production. These coordinated immunometabolic effects reduce systemic inflammation and may contribute to the attenuation of HS pathogenesis. Image credits: Declan C. McCann. Created using Microsoft PowerPoint. No AI-assisted tools were used. NLRP3, nucleotide-binding oligomerization domain (NOD)-like receptor protein 3; IL, interleukins; NF-κB, nuclear factor kappa-light-chain-enhancer of activated B cells; TLR4, toll-like receptor 4; TNF-α, tumor necrosis factor-alpha

Existing evidence supports the notion that SGLT2i targets multiple inflammatory immunometabolic pathways central to the pathogenesis of HS. Through the suppression of the NLRP3 inflammasome, the reduction of inflammatory cytokines, modulating macrophage polarization, and decreasing metabolic dysfunction, SGLT2i provides a potential strategy for the management of HS [17,18]. Despite this, direct clinical studies evaluating SGLT2i in patients with HS remain limited; further research is needed to determine whether these mechanistic benefits translate into true clinical improvement in HS severity. Figure 2 illustrates a proposed immunometabolic framework by which SGLT2i may attenuate inflammation in HS.

Evidence supporting SGLT2i use in other inflammatory diseases

Psoriasis

**Figure 3:**
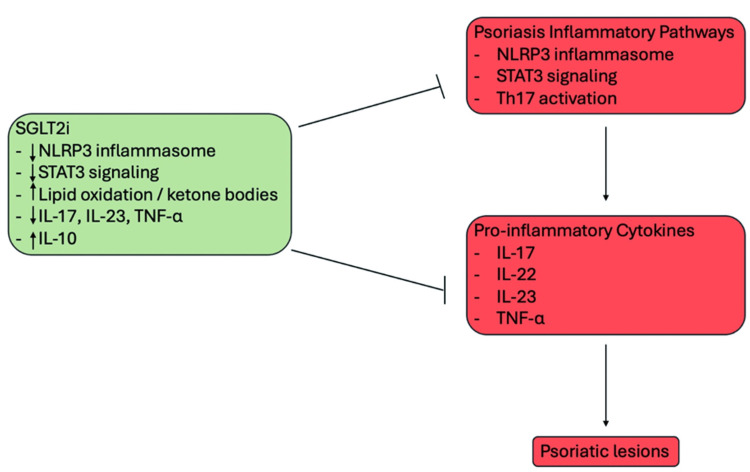
Potential mechanism of SGLT2i-mediated reduction in psoriatic inflammation. The figure illustrates the proposed pathways of how SGLT2is may attenuate psoriatic inflammation, including the inhibition of NLRP3 inflammasome activity, the suppression of STAT3 signaling, the modulation of Th17-associated cytokines, and the upregulation of IL-10 (green, effects of SGLT2is; red, inflammatory pathway in psoriasis). Image credits: Declan C. McCann. Created using Microsoft PowerPoint. No AI-assisted tools were used. NLRP3, nucleotide-binding oligomerization domain (NOD)-like receptor protein 3; IL, interleukins; NF-κB, nuclear factor kappa-light-chain-enhancer of activated B cells; TLR4, toll-like receptor 4; TNF-α, tumor necrosis factor-alpha; STAT3, signal transducer and activator of transcription 3; Th17, T helper 17 cell; SGLT2i, sodium-glucose cotransporter-2 inhibitor

Psoriasis is another immune-mediated chronic inflammatory skin disorder, characterized histologically by acanthosis, hypogranulosis, parakeratosis, and the dilation of vessels in the papillary dermis, causing visible erythema and a dense inflammatory infiltrate [23]. Its pathogenesis involves multiple inflammatory mediators, including the NLRP3 inflammasome, signal transducer and activator of transcription 3 (STAT3) signaling pathways, TNF, Th17 cells, IL-22, IL-23, and particularly IL-17 [24,25]. As previously mentioned, SGLT2is have been shown to suppress NLRP3 inflammasome activity by favoring ketone body production and lipid oxidation, which may reduce the levels of downstream cytokines such as IL-17 and IL-22 via Th17 cells [17,18,20]. Additionally, canagliflozin, an SGLT2i, has been shown to inhibit both the NLRP3 inflammasome and STAT3 signaling pathways, further suggesting a potential therapeutic role in inflammatory conditions driven by these pathways, including psoriasis [26,27]. Preclinical evidence further supports this possibility. In an imiquimod-induced mouse model of psoriatic-like dermatitis, canagliflozin treatment significantly reduced cutaneous concentrations of several inflammatory mediators, including IL-17, IL-23, and TNF-α, while increasing the anti-inflammatory IL-10 [28]. Figure 3 illustrates the proposed mechanisms by which SGLT2i may attenuate psoriatic inflammation through the modulation of STAT3 signaling, Th17-associated cytokines, and controlled NLRP3 inflammasome.

Alopecia Areata

Alopecia areata (AA) is an autoimmune, non-scarring hair loss disease with the preservation of the hair follicle [29]. Its pathogenesis is associated with the loss of immune privilege. In healthy individuals immune privilege consists of an anti-inflammatory environment around the hair follicle due to the lessened expression of major histocompatibility complex (MHC) class I and II molecules, as well as the high expression of natural killer (NK) cell inhibitors, while in individuals with alopecia areata, this loss of immune privilege is characterized by an increase in MHC molecules, a decrease in inhibitory signals, and the increased attack of the hair follicle by NK cells and CD8+ cells [29]. In addition to the loss of immune privilege, AA is associated with immunohistochemical staining showing increased levels of NLRP3, caspase-1, and IL-1β in the outer root sheath of hair follicles, which are all involved in chronic inflammatory signaling and follicular damage [19,29,30]. Similar to HS and other inflammatory diseases, the NLRP3 inflammasome has been suggested to contribute to both the onset and aggravation of AA in mouse model studies exploring how the reduction of NLRP3 signaling prevents AA development [31]. These studies additionally found that the inhibition of NLRP3 induced hair regrowth and inhibited inflammatory cell infiltration [31]. Given that SGLT2i has demonstrated the capability to attenuate the NLRP3 inflammasome in other diseases with similar mechanisms, its therapeutic effects may be explored in patients with AA [18].

Discussion

Conceptual Role of SGLT2i in HS

Our current understanding of HS indicates that it is a multifactorial disease involving chronic immune activation, follicular occlusion, and fibrosis. With that being said, it is unlikely that the modulation of metabolic inflammation alone would control the disease in moderate-to-severe cases of HS, especially in patients with existing structural disease [1,7,19]. The current treatment of HS requires multiple therapies, suggesting that any potential use of SGLT2i would be more effective as a combination therapy than as monotherapy [3,4].

In this context, SGLT2i is conceptualized as adjunctive therapy, specifically for patients with HS and metabolic dysfunction, such as obesity, metabolic syndrome, or insulin resistance [6,8,15,21,22]. It has been extensively studied that HS is associated with poor metabolic profiles, including increased fat mass, high rates of insulin resistance, and dysfunctional adipokine signaling. As previously discussed, there is a potential mechanistic link between these processes and the pathogenesis of HS, rather than mere association with the disease [6,8,21,22]. By targeting the metabolic aspect of HS pathogenesis, SGLT2is show promise as an adjunct in patients with metabolic comorbidities, alongside current treatments that target downstream inflammation.

Safety Considerations Relevant to Dermatology

While SGLT2is demonstrate promising therapeutic effects for HS and other inflammatory skin conditions, their safety profile must be carefully considered. One of the most serious adverse effects reported with SGLT2i usage is Fournier’s gangrene (FG), a rare type of necrotizing fasciitis involving the genital and perineal regions [32]. FG is characterized by a spread across fascial planes of the urogenital and anogenital regions, resulting in vascular occlusion, ischemia, and tissue necrosis [33]. Between 2013 and 2019, 55 cases reported FG in association with SGLT2i use, most commonly occurring in older men with comorbidities, such as type 2 diabetes mellitus (T2DM) and peripheral vascular disease [34]. Although an exact pathophysiology linking the two has not been found, one hypothesis stands that drug-induced glucosuria creates favorable conditions for urogenital infections, a risk factor for FG development [32]. Clinically, if a patient on an SGLT2i presents with genital tenderness, erythema, swelling, or pain, they should be assessed for FG [35]. Management may require hospitalization, the discontinuation of the SGLT2i, broad-spectrum antibiotic treatment, and the removal of necrotic tissue [34,35].

In addition to FG, several cutaneous adverse drug reactions (CADRs) have been associated with SGLT2is. A literature review compiled 37 papers identifying CADRs such as fixed drug eruptions, drug-induced pruritus, and acute febrile neutrophilic dermatosis, although fixed drug eruptions were the most common CADR found. An increased incidence of psoriasis has also been observed in patients with diabetes and renal diseases after beginning SGLT2i therapy. However, because most of these findings come from patients with T2DM, it is difficult to determine whether these CADRs are due to SGLT2is themselves or to patients’ underlying health conditions, as T2DM predisposes patients to skin and soft tissue infections. The aforementioned CADRs are managed using topical corticosteroids, antihistamines, and the discontinuation of the SGLT2is [36].

To prioritize patient safety and minimize complications, weighing these potential complications in populations at a higher risk of developing CADRs is key when determining whether SGLT2i use is indicated for inflammatory skin disorders.

Phenotype-Directed Approach

The phenotype-directed approach may be most appropriate for patients refractory to standard therapies [3,4]. These patients may have low-grade background inflammation due to their metabolic dysfunction, which sustains an immunometabolic environment that exacerbates cutaneous inflammation despite standard treatment [6,8,15,21,22]. Therefore, metabolically dysfunctional populations may represent the subgroup most likely to benefit from SGLT2 inhibition, rather than all individuals with HS.

Combination Therapy With Biologic Agents

**Figure 4:**
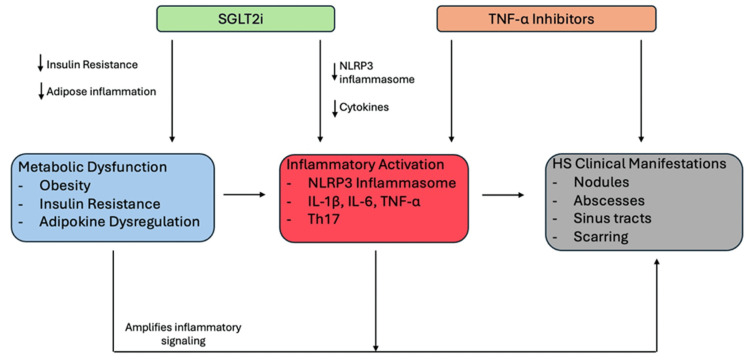
Integrated immunometabolic model of HS and therapeutic targets. Metabolic dysfunction (blue), including obesity, insulin resistance, and adipokine dysregulation, promotes inflammatory activation (red) via the NLRP3 inflammasome, cytokines, and Th17 signaling. These processes drive HS clinical manifestations (grey) of nodules, abscesses, sinus tracts, and scarring. SGLT2i (green) acts upstream by improving insulin resistance and reducing adipose inflammation while also inhibiting NLRP3 inflammasome activity and downstream cytokine production. On the other hand, TNF-α inhibitors target downstream cytokine activity. This model demonstrates the potential for combination therapies targeting both upstream and downstream mechanisms. Image credits: Declan C. McCann. Created using Microsoft PowerPoint. No AI-assisted tools were used. NLRP3, nucleotide-binding oligomerization domain (NOD)-like receptor protein 3; IL, interleukins; TNF-α, tumor necrosis factor-alpha; Th17, T helper 17 cell; SGLT2i, sodium-glucose cotransporter-2 inhibitor; HS, hidradenitis suppurative

There is potential complementarity between SGLT2i and existing biologic therapy. TNF-α inhibitors are one of the most common treatments for HS today. These biologics typically target downstream inflammation at the point of cutaneous inflammation, whereas SGLT2is may act further upstream by reducing hyperinsulinemia, modulating fat inflammation, shifting metabolism to lipid oxidation, suppressing the NLRP3 inflammasome, and improving insulin sensitivity [4,13-15,17-19]. Despite cytokine blockade with current therapeutics for HS, the upstream signaling of IL-1β/NLRP3 inflammasome signaling is still amplified in patients with HS. By using combination therapy with current biologics and SGLT2is, both upstream and downstream inflammation could theoretically be blocked, thereby increasing therapeutic effect and further supporting the use of SGLT2is as adjunct immunometabolic modulators rather than monotherapy [4,6,8,15,21]. Figure 4 integrates these pathways into a potential therapeutic model, highlighting SGLT2i targeting upstream immunometabolic pathways and TNF-α inhibitors acting on downstream inflammatory signaling in HS.

Integration in Multidisciplinary Care

SGLT2is’ best clinical fit would most likely be within a multidisciplinary mode of care, particularly at the intersection of dermatology and endocrinology [6,8,10,17,21,22]. As discussed, the patients most likely to benefit from SGLT2 inhibition are those with coexisting metabolic dysfunction. Thus, assessing endocrine markers may help identify which patients are most appropriate for treatment with these medications.

Limitations and Current Barriers

Although there is a strong mechanistic rationale for SGLT2is in inflammatory dermatological conditions such as HS, many barriers currently limit their use in this setting [3,4,17,18]. The direct clinical evidence for SGLT2 inhibition in HS is extremely limited; our rationale is based on a convergence of several mechanistic pathways between HS inflammation and SGLT2 inhibition, rather than on true disease-specific trial data [4,17,18]. All use of SGLT2i today for the treatment of HS would be considered off-label.

Their use specifically as a monotherapy is limited due to the complex presentation of HS. There is sound reasoning that the use of SGLT2i would attenuate the inflammatory response that fuels the pathophysiology of HS. However, it is unlikely that SGLT2 inhibition will reverse fibrosis or chronic tissue destruction that has already occurred in advanced disease processes. As a result, their greatest potential lies in adjunct therapy for particular patient populations, such as those with cardiometabolic disorders, in which proper immunometabolic signaling may play a positive role in controlling and treating disease [1,18,19].

Future Directions and Research Priorities

With growing evidence supporting HS as a systemic inflammatory disorder closely linked to metabolic dysfunction, this raises important questions about whether modulating metabolic pathways can alter disease activity. While current evidence indicates that SGLT2i use can affect several pathways involved in HS, including inflammasome activation, cytokine signaling, and insulin resistance, clinical studies examining these effects in patients with HS remain limited.

Future studies should focus on the use of SGLT2i as either monotherapy or an adjuvant in patients with HS. Included in these studies should be measurements of lesion count, disease severity scores, and measurements of metabolic markers, such as inflammatory cytokines and inflammasome activity. Collecting these measurements would allow for the total effects of SGLT2i use to be better understood and defined. Additionally, similar studies should be conducted on individuals who have comorbidities, including obesity, metabolic syndrome, or insulin resistance. Exploring the effects of SGLT2i in these specific populations may clarify how these comorbidities may affect treatment and potentially highlight certain populations in which SGLT2i use may be more beneficial.

Further experimental research is also needed to better understand how immunometabolic signaling affects HS development and progression. Future studies should investigate the effects of modulating macrophage polarization, adipokine regulation, keratinocyte proliferation, and NLRP3 inflammasome activity. This would allow for a deeper understanding of how metabolic dysfunction impacts the chronic state of inflammation seen in HS. Clarifying this may explain why patients with HS often have comorbid conditions and provide insight into how developing therapies can target both the metabolic and inflammatory components of the disease.

As the metabolic involvement in HS pathogenesis becomes more widely recognized, targeting the underlying metabolic dysfunction could be a promising new strategy for treating the disease. Currently available therapies only aim to suppress inflammatory mediators, not the metabolic factors upstream of them. Research should continue to investigate treatments that modulate these metabolic pathways, including SGLT2is, as they may offer new treatment options, especially for patients who have been resistant to current therapies or have comorbid metabolic conditions.

## Conclusions

Although the pathophysiology of HS development is not fully understood, the role of metabolic dysfunction is increasingly recognized. With this growing recognition, new therapies targeting metabolic dysfunction, including SGLT2i, have become a promising area of investigation, as they may modulate the immunometabolic pathways implicated in HS. This suggests that SGLT2i may be a potential therapeutic option in HS; however, the clinical data supporting this remain limited. Future studies are necessary to determine whether these therapies significantly improve disease outcomes in patients with HS and metabolic comorbidities.
